# Systemic Inflammatory Biomarkers and Cancer-Associated Cachexia: A Narrative Review

**DOI:** 10.3390/curroncol33080483

**Published:** 2026-08-15

**Authors:** Shawna Landon, Jaclyn M. Hall, Saunjoo L. Yoon

**Affiliations:** 1 College of Nursing, University of Florida, Gainesville, FL 32610, USA; yoon@ufl.edu; 2 College of Medicine, University of Florida, Gainesville, FL 32610, USA; jaclynha@ufl.edu

**Keywords:** cancer-associated cachexia, IL-6, TNF-α, C-reactive protein, neutrophil-to-lymphocyte ratio, albumin, systemic inflammation, biomarker, prognosis, muscle wasting

## Abstract

Cancer-associated cachexia (CAC) is a multifactorial syndrome characterized by systemic inflammation, muscle wasting, and progressive functional decline, affecting up to 80% of patients with advanced cancer. The inconsistent diagnostic criteria limit comparability across studies and hinder translation into clinical practice. Inflammatory biomarkers, including interleukin-6 (IL-6), tumor necrosis factor-alpha (TNF-α), C-reactive protein (CRP), neutrophil-to-lymphocyte ratio (NLR), and serum albumin, have been studied as markers of the presence, severity, and progression of CAC. This review synthesizes evidence linking these five biomarkers to key manifestations of CAC, including weight- loss, muscle depletion, fatigue, quality of life, and survival. A structured search of PubMed, Embase, and Web of Science studies published between 2000 and 2025, yielding 20 articles for final synthesis to evaluate biomarker patterns and their potential utility for early detection and risk stratification. IL-6 and CRP show consistent associations with muscle wasting and systemic inflammatory burden. Albumin and NLR demonstrate enhanced prognostic value when incorporated into composite indices such as the Cachexia index. TNF-α remains mechanistically relevant but shows limited predictive utility when assessed independently. CRP, Albumin, and NLR are derived from routine, low-cost tests, whereas IL-6 and TNF-α require specialized cytokine assays. Future research is warranted to standardize diagnostic criteria and validate biomarker thresholds to develop an accessible, multi-biomarker panel for improved detection and monitoring of CAC.

## Simple Summary

Cancer-associated cachexia is a multifactorial syndrome characterized by systemic inflammation, weight loss, and poor nutritional intake secondary to anorexia, affecting up to 80% of patients with advanced cancer. Despite its profound impact on survival and quality of life, inconsistent diagnostic criteria and heterogeneity in measurement across research studies limit its translation into routine clinical practice. Inflammatory biomarkers, including interleukin-6, tumor necrosis factor-alpha, C-reactive protein, the neutrophil-to-lymphocyte ratio, and serum albumin, have been widely investigated for their ability to reflect the presence, severity, and progression of cachexia. This review aimed to synthesize current evidence on these key biomarkers to clarify their clinical utility and to support efforts toward standardized assessment, early recognition, and improved monitoring of cancer-associated cachexia.

## 1. Introduction

Cancer remains a leading cause of death globally, accounting for approximately 10 million deaths each year [1]. Cancer-associated cachexia (CAC), as one of the most debilitating complications, is estimated to affect up to 80% of patients with advanced-stage cancers and is estimated to account for 20–30% of cancer-related deaths [2,3]. It is particularly prevalent in gastrointestinal malignancies, including pancreatic, colorectal, and gastric cancers [2,4].

CAC is a complex metabolic syndrome characterized by progressive skeletal muscle loss, with or without fat loss, that cannot be reversed with standard nutritional support [5]. The progressive muscle wasting contributes significantly to morbidity and mortality in all stages of cancer by impairing treatment tolerance and accelerating clinical decline [5].

Unlike simple malnutrition, CAC arises from a multifactorial interplay of systemic inflammation, metabolic disruption, muscle wasting, and reduced nutritional intake, resulting in profound weakness, reduced quality of life, and decreased survival [3]. Central to this process is chronic systemic inflammation, driven largely by pro-inflammatory cytokines. Tumors release factors such as proteolysis-inducing factor (PIF) that activate NF-κB signaling pathways, prompting cells to produce more cytokines and chemokines and sustaining a self-perpetuating inflammatory state [6]. Despite its clinical significance, CAC is often underdiagnosed and undertreated. A major barrier is the lack of standardized diagnostic criteria, which complicates early identification and differentiation of related conditions such as general malnutrition or cancer-related fatigue [5,7]. Early detection of CAC is critical, as timely intervention may help preserve muscle mass, improve functional status, and potentially prolong survival [5,7]. Identifying reliable biomarkers for CAC and its severity may facilitate earlier diagnosis, guide treatment decisions, and improve patient outcomes. For each biomarker discussed below, we consider both its mechanistic contribution to the inflammatory, metabolic, and nutritional processes underlying CAC and its demonstrated utility as a clinical indicator of cachexia severity or prognosis.

Systemic inflammation is increasingly recognized as a key driver of CAC. Although numerous biomarkers have been investigated in CAC [7], this review focuses more on IL-6, TNF-α, CRP, NLR, and serum albumin, which are consistently and most commonly evaluated across the literature reviewed. IL-6 and TNF-α drive muscle catabolism, the breakdown of skeletal muscle protein, by activating intracellular pathways such as NF-κB and the ubiquitin–proteasome system, which tag muscle proteins for degradation, while also sustaining the systemic inflammatory signaling that propagates the cachectic state [6,7]. Biomarkers such as IL-1β, IL-18, and GDF-15 (Growth Differentiation Factor 15) are less commonly mentioned and have been increasingly assessed in recent years. IL-1β acts upstream of this cascade: it is released via caspase-1/NLRP3 inflammation activation triggers NF- κB signaling and induces IL-6 production, thereby amplifying the same catabolic and acute-phase pathways that drive muscle wasting and elevate CRP [8]. IL-18, structurally related to IL-1β and also processed through the NLRP3/caspase-1 pathway, contributes a complementary role by regulating energy metabolism, including sensitivity in adipose tissue, and its deficiency in human and animal models is associated with excess food intake, obesity, and insulin resistance, suggesting a role in the metabolic food dysregulation underlying cachexia [8]. GDF-15, a member of the TGF-B, has drawn increasing attention for its strong association with cancer-associated anorexia and metabolic dysregulation in cachexia [9]. GDF-15 signals through a receptor complex, GFRAL and its co-receptor RET, that is highly localized to the hindbrain, and it levels can rise substantially in advanced cancer, correlating with anorexia and weight loss; in a phase 2 clinical trial, an antibody blocking GDF-15 signaling significantly increased body weight, muscle mass and appetite in patients with cancer cachexia [10].

CRP, a liver-produced acute-phase protein synthesized in response to IL-6, rises as a downstream marker of this inflammatory activity and reflects the overall systemic inflammatory burden [6]. Elevated CRP has been associated with greater cachexia severity and poorer prognosis in advanced cancer [11,12,13]. NLR is a readily accessible marker derived from routine blood counts that captures the balance of neutrophil-driven inflammation and lymphocyte-mediated immune function [11,12]. Albumin, a negative acute-phase protein, reflects both nutritional status and inflammation [12,13]. Collectively, these biomarkers offer insights into the biological and clinical dimensions of CAC.

CAC is commonly assessed using indicators such as involuntary weight loss, skeletal muscle wasting, reduced physical function (e.g., stair-climb power, handgrip strength), and symptoms like fatigue, anorexia, and general weakness [5]. Although each marker reflects a distinct aspect of the inflammatory response, together they capture the same underlying systemic inflammation that drives CAC, which makes them informative both individually and in combination as indicators of disease severity [13,14]. Composite indices such as the Cachexia Index (CXI), which integrates skeletal muscle index (SMI), serum albumin, and NLR, capture the inflammatory and nutritional dimensions of CAC and have been validated as prognostic tools [15]. Lower CXI scores specifically are associated with more advanced tumor stage, higher inflammatory markers, poorer nutritional status, and worse survival [12,15].

This narrative review synthesizes the current evidence linking key systemic inflammatory biomarkers to CAC and its severity in adults with cancer. It integrates findings across studies, highlights methodological and conceptual limitations, and evaluates the clinical utility of the biomarkers for risk stratification and management of CAC.

## 2. Materials and Methods

### Search Strategy and Information Sources

**Figure 1:**
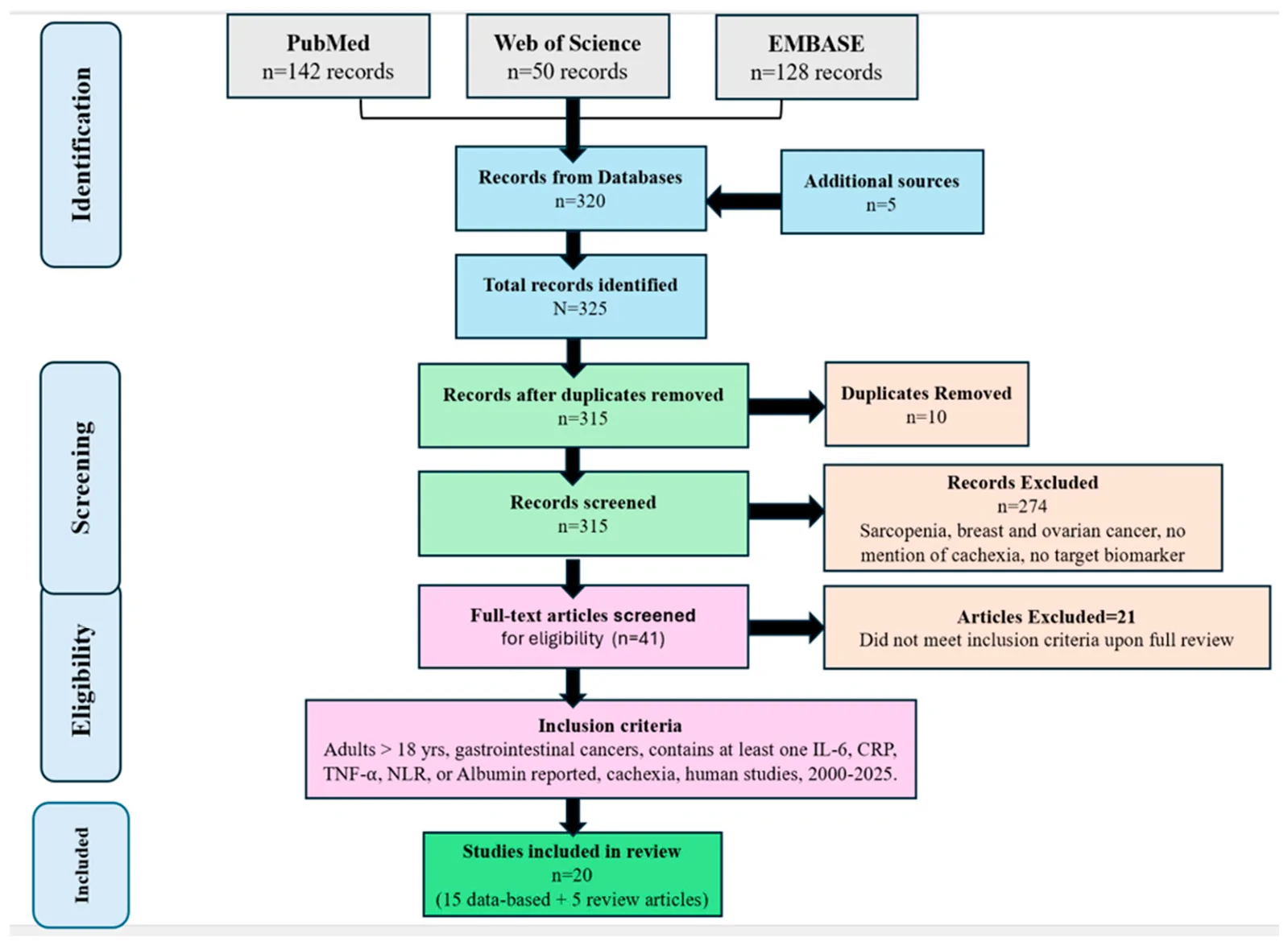
PRISMA Flowchart for selection of Eligible Sources.

This narrative review adopted a systematic approach to the literature search and study selection process to improve transparency and reduce selection bias, while retaining the interpretive and non-exhaustive characteristic of a narrative review format. This independently conducted narrative review examined the relationship between systemic inflammatory biomarkers (IL-6, CRP, TNF-α, NLR, and albumin) and CAC in adults. A structured search was performed in PubMed, Web of Science, and Embase for studies published between January 2000 and September 2025. In addition to the structured database search, a supplementary search of recent literature was conducted to identify emerging inflammatory biomarkers relevant to CAC that were not captured by the original search terms. The broader timeframe was selected to capture foundational research following the first formal recognition of cachexia as a clinical syndrome [5]. To ensure the synthesis reflects the most current clinical evidence, only empirical studies reporting patient data (e.g., clinical trials, observational studies) published between 2017 and 2025 were included in the final synthesis. Search terms combined with Medical Subject Headings (MeSH) and keywords related to cancer, cachexia, and biomarkers. Core terms included: (“cancer” AND “cachexia” AND “biomarker”), along with specific biomarkers: (“IL-6” OR “interleukin-6” OR “C-reactive protein” OR “CRP” OR “TNF-alpha” OR “tumor necrosis factor-alpha” OR “neutrophil-to-lymphocyte ratio” OR “NLR” OR “albumin”). Additional outcome-related terms such as “severity,” “progression,” “prognosis,” and “inflammatory marker”) were included to improve the relevance of articles addressing the clinical implications of biomarkers in CAC. See Figure 1 for more details.

## 3. Results

Across the 15 data-based studies and 5 review articles, IL-6, CRP, TNF- α, NLR, and albumin demonstrated associations with CAC, though they varied in consistency, clinical utility, and strength of evidence. Collectively, these biomarkers reflect the multifactorial nature of CAC, capturing inflammatory, nutritional, and functional dimensions. The sections below examine each biomarker (Table 1).

### 3.1. Interleukin-6 (IL-6)

IL-6, a pro-inflammatory cytokine involved in immune signaling and the acute-phase response, emerged as the most frequently studied inflammatory biomarker across the reviewed literature, with eight of the fifteen data-based articles evaluating its role in CAC [11,12,13,14,17,21,25,27]. Elevated IL-6 levels, commonly measured using enzyme-linked immunosorbent assay (ELISA) or multiplex cytokine assays, are associated with muscle wasting, systemic inflammation, reduced physical function (e.g., performance status assessed using scales such as the Eastern Cooperative Oncology Group [ECOG], and handgrip strength), increased fatigue, and decreased quality of life across diverse cancer types [14,17]. Higher baseline IL-6 (≥18 pg/mL) was associated with reduced serum albumin and shorter survival in patients with advanced cancer [14], and circulating IL-6 was significantly elevated in cachectic patients relative to non-cancer controls (*p* ≥ 0.009), a group that also exhibited lower stair-climb power, hand grip strength, and appetite alongside greater fatigue [17].

Clinical and mechanistic evidence support IL-6 as a central mediator of CAC. Expression in preclinical models drives muscle degradation and systemic inflammation [14,27]. Clinical data demonstrated that IL-6 levels were significantly higher in cancer patients with cachexia than in non-cachectic patients and controls. Elevated IL-6 negatively correlated with stair-climb power and upper body strength [17] and was a predictor of reduced lean muscle mass and increased resting energy expenditure [12].

IL-6 is linked to worsening fatigue and gut-mucosal inflammatory response that may initiate or perpetuate CAC [21,25]. In a review of anti-inflammatory strategies, this cytokine was identified as a central mediator in tumor–host interactions, supporting its role as a therapeutic target [14]. Higher IL-6 levels were associated with lower muscle mass, worse appetite, and poorer overall quality of life, including increased fatigue, weakness, and decreased physical independence [12]. Elevated IL-6 levels were consistently associated with worsening cachexia indicators, spanning both metabolic (e.g., muscle catabolism, impaired protein synthesis, altered energy expenditure) and symptomatic domains (e.g., fatigue, anorexia, reduced physical function) [11]. Strengths of the IL-6 include the integration of biological measures (e.g., serum IL-6 levels, C-reactive protein, albumin) and objective physical function measures (e.g., handgrip strength, stair climb power) across diverse cancer types (e.g., pancreatic, gastric, colorectal) and disease stages (early to advanced) [21,25].

However, inconsistencies in cachexia definitions, measurement protocols, and heterogeneity in assay methods (e.g., ELISA vs. multiplex assays) may limit reproducibility and comparability across studies [11]. Gaps include a lack of longitudinal studies assessing IL-6 levels during CAC progression and treatment [27]. Despite its biological relevance, IL-6 remains an underexplored therapeutic target in clinical trials, with most current research focusing on observational associations [14]. More standardized protocols and consistent cachexia criteria could enhance the translational potential of findings related to IL-6 and other inflammatory biomarkers [14,27].

### 3.2. C-Reactive Protein (CRP)

CRP was assessed in 11 of the 15 data-based studies, making it one of the most consistently reported biomarkers in CAC. CRP is a liver-derived acute-phase protein induced primarily by IL-6 and serves as a widely used marker of systemic inflammation [16]. In cancer cachexia, elevated CRP reflects this inflammatory state and is associated with anorexia, weight loss, and muscle wasting [16]. Across gastrointestinal cancers (e.g., pancreatic, gastric, and colorectal cancers), elevated CRP and systemic inflammation were associated with greater cachexia burden, including poorer quality of life [21]. A large cohort of 1702 advanced cancer patients receiving palliative care demonstrated that higher CRP strata were associated with progressively worsening anorexia, fatigue, weight loss, and dependence in activities of daily living [16].

CRP also features in diagnostic frameworks for cachexia, such as the Asian Working Group for Cachexia (AWCG) criteria, in which elevated CRP (>5 mg/L) is one of the three diagnostic components [15]. The CXI, a composite prognostic marker integrating skeletal muscle index, serum albumin, and neutrophil-to-lymphocyte ratio, was validated against the AWGC criteria [15]. In gastric cancer, low CXI independently identified patients meeting AWGC-defined cachexia and was associated with poorer health-related quality of life on the EORTC QLQ-C30 [15]. Together, these findings support CRP as a useful marker of systemic inflammation in cachexia, particularly when incorporated into composite indices or diagnostic criteria. Because CRP levels rise in response to systemic inflammation, they serve as a dynamic marker of CAC progression and severity.

Mechanistic links further highlight the biological role of CRP. Cachectic patients with colorectal cancer had higher CRP and CRP/albumin ratios along with gut barrier disruption, immune cell infiltration, and altered cytokine expression, suggesting systemic CRP elevations may partly reflect intestinal immune activation associated with tumor-induced inflammation and mucosal damage in cancer cachexia [21]. Other studies have reinforced these links, showing that high CRP levels commonly coincide with hypoalbuminemia (reflecting poor nutritional and inflammatory status), anemia (associated with fatigue and reduced oxygen delivery), increased cytokines (driving force of systemic inflammation), and greater cachexia-related symptom burden [12,14]. The findings underscore CRP’s dual role as both a marker of systemic inflammation and a window into the pathophysiology of cachexia. Despite its strengths, CRP is nonspecific and can be elevated by infection, treatment effects, or comorbidities, and cutoff definitions vary across studies [14]. Most research captures CRP at a single time point rather than longitudinally, limiting insight into its dynamic role [11]. While CRP was associated with symptom burden and functional decline in palliative care patients, its relationship with skeletal muscle mass and activities of daily living was not directly investigated, representing a gap in understanding CRP’s full clinical utility at the bedside [24]. The cross-sectional design of several studies limits the ability to establish a causal relationship between CRP levels and progression of cachexia [24]. Future work should standardize CRP thresholds, test if CRP changes predict cachexia onset or progression, and evaluate whether anti-inflammatory interventions that reduce CRP improve outcomes in patient with CAC. Elevated CRP reliably signals greater cachexia burden and worse prognosis, especially when paired with nutritional and functional markers [15,27].

### 3.3. Tumor Necrosis Factor-Alpha (TNF-α)

TNF-α was examined in five of the fifteen data-based studies and has long been recognized as a central mediator in the inflammatory processes driving cancer cachexia. Elevated TNF-α levels are correlated with weight loss, reduced muscle mass, anorexia, fatigue, and systemic inflammation, making it one of the earliest cytokines implicated in CAC [14,27]. In clinical studies, elevated TNF-α levels have been associated with declines in nutritional markers such as serum albumin and hemoglobin, underscoring its link to metabolic dysfunction and disease severity [13,17].

Mechanistically, TNF-α promotes proteolysis and lipolysis through activation of intracellular signaling pathways—such as the NF-κB and ubiquitin–proteasome pathways—that degrade muscle proteins and fat stores, contributing to the metabolic derangements of cachexia [14]. TNF-α and IL-6 interact to promote inflammatory signaling and drive muscle degradation in cachexia [14,27].

This redundancy makes it difficult to isolate its precise mechanistic contributions, yet elevated levels consistently coincide with lower albumin, higher CRP, and greater fatigue, reinforcing its value as a biomarker of cachexia severity [13,17]. TNF- α has typically been evaluated as part of a broader panel of inflammatory and metabolic biomarkers rather than in isolation [14]. Future research should prioritize integrated, multi-target approaches that address the complexity of cachexia rather than single-cytokine interventions [13,14].

### 3.4. Neutrophil-to-Lymphocyte Ratio (NLR)

The NLR emerged as a frequently studied biomarker in eight studies examining its relevance in CAC [14,15,16,19,20,21,24,28]. As an accessible indicator of systemic inflammation, NLR is derived from a standard complete blood count (CBC), a routine lab test performed in most clinical settings. Although not always reported directly, NLR can be easily calculated and is commonly assessed alone or within composite indices such as the CXI or Naples Prognostic Score (NPS), which is a categorical score combining albumin, total cholesterol, NLR, and lymphocyte-to-monocyte ratio to reflect inflammatory and nutritional status, differing from the CXI in that it does not incorporate skeletal muscle mass [23,28]. Across gastric, colorectal, lung, and pancreatic cancer populations, elevated NLR was consistently associated with greater inflammation, reduced skeletal muscle mass, diminished quality of life, increased complications, and shorter overall survival [14,15,16].

Several studies incorporated NLR into multi-parameter tools to improve prognostic accuracy. NLR was integrated in the CXI and was independently associated with CAC and lower health-related quality of life (HRQoL) in gastric cancer patients, even after adjusting for clinical risk factors [15]. High NLR values were also associated with clinical indicators of CAC severity, including ≥5% weight loss, low skeletal muscle index (SMI), and reduced physical function [20]. Similarly, in the NPS, higher scores indicated worse inflammation and nutritional status, and were associated with increased cancer incidence and cancer-specific mortality in a large community cohort [28]. NLR also emerged as a strong predictor of cachexia-related outcomes in patients with advanced colon, lung, or prostate cancer [18].

In gastric cancer, elevated NLR predicted lower CXI scores, which were associated with advanced tumor stage, elevated inflammatory markers (CRP and IL-6), poor nutritional status (low albumin and prealbumin), increased postoperative complications, such as pulmonary infections and significantly worse overall survival [12]. In pancreatic cancer, high NLR was associated with rapid muscle wasting and poorer prognosis [19,20]. Reviewed studies showed strengths such as large, diverse enhancing populations, from community cohorts to surgical patients, and enhancing generalizability [12,15,28].

However, limitations were evident. Studies used different cutoff values for high NLR, such as 3.15, which may affect consistency and interpretation [18]. All eight studies evaluating NLR were either retrospective or cross-sectional in design, limiting the ability to infer causality or assess temporal changes in NLR [14,15,16,19,20,21,24,28]. While NLR is recognized as a marker of inflammation, elevated NLR reflects a chronic inflammatory state that promotes muscle protein degradation, impairs muscle regeneration, and increases oxidative stress, factors that together contribute to impaired protein synthesis and enhance protein breakdown in cancer-associated cachexia [28].

In summary, NLR is a promising, low-cost biomarker measured as the ratio of neutrophils to lymphocytes in the blood, and it is associated with cachexia severity and outcomes across multiple cancer types [12]. While its inclusion in indices like CXI and NPS improves prognostic value, more research is needed to define optimal cutoffs, track changes over time, and assess whether interventions that lower NLR, such as inflammatory or immunomodulatory, can affect progression or improve patient outcomes [18,19].

### 3.5. Albumin

Albumin was among the most frequently investigated biomarkers in the reviewed literature, appearing in 12 of the 15 data-based studies. Consistently, serum albumin levels were significantly lower in cachectic patients compared to weight-stable patients, reflecting both poor nutritional status and systemic inflammation [14,21,22,25]. Albumin is a negative acute-phase protein that decreases during inflammation [11]. Although it is a useful indicator of CAC severity, its levels can also be influenced by liver function, nutritional intake, and fluid balance [11].

Several studies incorporated albumin into composite scores to improve prognostic accuracy. The CXI, combining albumin, SMI, and NLR, was linked to poorer survival and reduced physical function in gastrointestinal and lung cancers [12,20,23]. Similarly, the NPS is not a cachexia-specific index score but was associated with increased mortality risk in cancer survivors, likely reflecting the inflammatory and nutritional disturbances common in CAC [13,28].

Low albumin is also correlated with higher CRP levels and worsened survival outcomes, reinforcing its value as a marker of both inflammation and cachexia severity [22,25]. While albumin remains widely accessible, its specificity is limited by factors like hydration and liver function [13]. Still, its use of multidimensional prognostic tools enhances its clinical relevance. Further research should explore whether improving nutritional status and reducing inflammation, which may increase albumin levels, can influence the progression of cancer cachexia [27]. Recent evidence shows that low albumin levels, particularly when combined with elevated CRP and low BMI in the Prognostic Nutritional CRP Ratio (NCR), are strongly associated with reduced survival and lower quality of life in patients with cancer cachexia [27] (Table 2).

## 4. Discussion

This review identified IL-6, CRP, TNF-α, NLR, and albumin as five key inflammatory biomarkers associated with CAC severity. IL-6 and CRP demonstrate the most consistent association with CAC. Considered along the mechanistic-clinical axis outlined in the introduction, IL-6 and TNF-α are mechanistically central to the catabolic and inflammatory process driving CAC, yet their independent predictive value may be limited and more informative when evaluated alongside other biomarkers rather than in isolation [22]. In contrast, CRP, NLR, and albumin are less mechanistically specific but offer greater practical clinical utility: albumin, neutrophils, and lymphocytes are routinely obtained during clinical visits and are easy to incorporate into the calculation of composite indices, such as the CXI and NPS, and CRP demonstrates a consistent association with CAC severity as a readily accessible marker of systemic inflammatory burden.

Clinical translation is limited by heterogeneity of CAC definitions, biomarker thresholds, and study design. Currently, no single biomarker has been identified to define or diagnose CAC [6]. No single intervention has proven consistently effective in reversing the syndrome or preventing mortality [3]. Integrating inflammatory biomarkers into routine clinical assessment may enable earlier detection of cachexia, allowing for timely interventions that could mitigate symptom progression and improve patient outcomes, as well as more personalized management [6].

Future research should prioritize standardizing diagnostic criteria, identifying reliable biomarkers for diagnosing CAC, establishing validated biomarker thresholds to assess CAC severity, and conducting longitudinal studies to examine the association of these biomarkers with temporal changes and treatment outcomes. Advancing Multi-biomarker-guided interventions and mechanistic approaches will be critical to improving clinical outcomes in CAC care [26]. In addition, biomarkers such as IL-1β, IL-18, and GDF-15, though studied in the CAC literature to date, warrant further investigation given their mechanistic involvement in the inflammatory and metabolic pathways driving cachexia; clarifying their independent and combined contributions may reveal additional layers of complexity in CAC pathophysiology and uncover new opportunities for bio-marker guided or therapeutic interventions. Notably, these biomarkers have not been consolidated into a single standardized blood panel for cancer cachexia. CRP, albumin, and NLR are available through clinical laboratory testing, whereas IL-6 and TNF-α require specialized cytokine assays that pose challenges for clinical application. Developing and validating a standardized, accessible CAC biomarker panel remains critically important.

## 5. Conclusions

Systemic inflammatory biomarkers are critical to provide information on severity and clinical forecast regarding cancer-associated cachexia. IL-6 and CRP demonstrated consistent associations with cachexia-associated symptoms, while albumin and NLR offer practical, low-cost utility for risk stratification. Regardless of its utility, no single biomarker is sufficient to evaluate cancer-associated cachexia. It warrants the development of a composite index that uses clinically available biomarkers to improve patients’ outcomes.

## Figures and Tables

**Table 1:** Evidence Summary: Systemic Inflammatory Biomarkers Associated with Cancer-Associated Cachexia Severity.

Authors	Study Design & Purpose	Participants	Methodology	Results
Amano et al. [16]	Secondary data analysis of a multicenter prospective cohort.	1702 adults with locally extensive or metastatic cancer.	CRP levels categorized into four groups. Eight symptoms and five ADL disabilities assessed at baseline using chi-square tests and ordinal logistic regression. Adjusted for age, gender, cancer site, metastatic disease, ECOG PS, chemotherapy, and care setting.	Higher CRP levels were significantly associated with greater symptom burden and ADL disabilities in advanced cancer patients. The very high CRP group showed the strongest associations with anorexia, fatigue, weight loss, and functional decline. Supports CRP as a relevant inflammatory biomarker in cancer cachexia progression.
Anderson et al. [17]	Cross-sectional study To evaluate functional performance, cytokines, and quality of life in men with and without cancer cachexia.	133 men total: 48 with cancer cachexia (CAC), 48 with cancer but no cachexia (CNC), and 37 healthy controls.	Fasting assessments of body composition via DEXA, resting energy expenditure via indirect calorimetry. Physical function assessed via stair climb power, handgrip strength, and 1RM muscle strength. Inflammatory biomarkers (IL-6, TNF), testosterone, and patient-reported quality of life assessed. ROC curves and multivariate regression used to identify functional impairment cut points.	CAC demonstrated significantly reduced lean mass, stair climb power, and upper body strength alongside elevated inflammatory cytokines (IL-6 and TNF) and relative energy expenditure. A stair climb power cut-point of 336 W (78% sensitivity, 77% specificity) best discriminated cachexia. Supports stair climb power and upper body strength as relevant functional impairment tools in cancer cachexia assessment.
Aro et al. [11]	Retrospective cohort study To examine associations between myosteatosis, systemic inflammation and survival in colorectal cancer patients.	222 colorectal cancer patients; majority male (53%) and overweight, obese (66.7%); 87% underwent surgery with curative intent.	Myosteatosis defined from preoperative CT scans at the L3 vertebral level using muscle density (Hounsfield units). Systemic inflammation assessed via NLR, serum CRP, and albumin. Multivariable binary logistic regression and Kaplan–Meier survival analysis with Cox regression performed.	Myosteatosis prevalence was 30%; associated with elevated NLR (*p* = 0.005), low albumin (*p* = 0.018), proximal tumor location, and serrated tumor morphology. Myosteatosis was associated with shorter overall survival in multivariable survival analysis. Supports myosteatosis as an inflammatory and cachexia-related marker in colorectal cancer.
Baracos et al. [7]	Narrative review To provide comprehensive overview of cachexia definition, epidemiology, mechanisms, diagnosis, and management.	No empirical data collected. Synthesis of existing literature across multiple cancer types.	Narrative synthesis of existing preclinical and clinical literature covering mechanisms of muscle and adipose tissue loss, pro-inflammatory cytokines (IL-6, TNF, IL-1), energy expenditure, diagnostic criteria, nutritional interventions, and pharmacological therapies. No primary data collection or statistical analysis performed.	Cachexia is a multifactorial syndrome driven by inflammation, reduced food intake, and metabolic dysregulation. Pro-inflammatory cytokines, IL-6 and TNF drive skeletal muscle catabolism. No approved drug therapies currently exist; nutritional support remains the primary treatment approach. Multimodal care targeting inflammation, nutrition, and physical activity is recommended as the optimal management strategy.
Barker et al. [18]	Retrospective cohort study To Investigate the association between elevated NLR and weight loss and cachexia in advanced cancer patients.	50 adults with newly diagnosed advanced colon, lung, or prostate cancer (stages III/IV).	NLR calculated from neutrophil and lymphocyte counts at diagnosis. Patients divided into above or below median NLR groups; body weight assessed at two time points. Cachexia defined as weight loss greater than 5%. Mann–Whitney U, chi-squared, and Kaplan–Meier analyses performed.	Weight loss was significantly greater in the above-median NLR group compared to the below-median group (*p* < 0.05). Cachexia prevalence was significantly higher in the above-median NLR group (44%) versus the below-median group (12%) (*p* < 0.05). Supports NLR as a readily available inflammatory biomarker associated with cachexia progression in advanced cancer.
Basile et al. [19]	Retrospective observational cohort study To examine the prognostic impact of early skeletal muscle mass loss on survival in advanced pancreatic cancer patients.	94 advanced pancreatic cancer patients; 55% male, 50% metastatic disease; 73% were sarcopenic at baseline.	Skeletal muscle index (SMI) measured from CT scans at the L3 vertebral level at baseline and after approximately 3 months. LSMM defined as SMI decrease greater than 10%. Inflammatory biomarkers assessed including NLR, albumin, and CRP. Cox regression, logistic regression, log-rank tests, and Kaplan–Meier survival curves used for statistical analysis.	Early LSMM ≥ 10% occurred in 21% of patients and was independently associated with worse overall survival (HR 2.16, *p* = 0.007) and progression-free survival (HR 2.31, *p* = 0.004) in multivariate analysis. NLR variation was significantly associated with early LSMM (OR 1.31, *p* = 0.010). Supports CT-based SMI monitoring as a more sensitive tool than weight loss alone for assessing cachexia progression in pancreatic cancer.
Cehreli et al. [14]	Prospective cohort study To examine whether inflammatory and nutritional serum markers predict chemotherapy outcomes in advanced non-small cell lung cancer (NSCLC).	25 adults with advanced stage NSCLC (stages 3B/4); 84%, 80% malnourished at baseline.	Nutritional status assessed by Subjective Global Assessment (SGA) and ECOG performance status. Serum albumin, CRP, and cytokines (IL-6, IL-1β, TNF-α) measured at baseline, 2 months, and 4 months by ELISA. GPS calculated from CRP and albumin levels. Wilcoxon and Friedman tests used for statistical analysis.	96% of patients had elevated CRP; 80% had abnormal GPS. Elevated CRP, IL-6, and TNF-α were associated with reduced albumin and survival. TNF-α decreased significantly after chemotherapy (*p* = 0.028). Findings support inflammatory and nutritional biomarkers as useful predictors of cachexia and survival in advanced NSCLC, though results are limited by a very small sample size.
Chen et al. [20]	Retrospective-controlled study Supporting evidence to examine IL-6 receptor inhibition via tocilizumab in cancer cachexia with systemic hyperinflammation.	40 adults with advanced cancer; 20 tocilizumab treatment; 20 matched controls.	TCZ group received a single intravenous dose of tocilizumab plus corticosteroids; control group received corticosteroids only. Outcomes assessed included NLR, CRP, albumin, prealbumin, handgrip strength, and weight change. Mann–Whitney U and chi-squared tests used for statistical analysis.	TCZ treatment significantly reduced NLR and CRP levels and improved albumin and prealbumin levels compared to controls. Supports CRP and NLR as dynamic markers of cachexia severity and progression. Findings limited by small sample size of 40 patients.
Costa et al. [21]	Cross-sectional study To examine gut barrier disruption as a source of systemic inflammation in cachectic versus weight-stable colon cancer patients.	45 adults with colorectal cancer patients; 25 cachectic, 20 weight-stable; no prior anticancer treatment.	Blood and colon tissue samples collected during surgery. Morphological, immunological, and molecular analyses performed using light microscopy, immunohistochemistry, flow cytometry, and Luminex xMAP multiplex cytokine assay. CRP, albumin, and hemoglobin measured from blood samples. Student’s *t*-test, Mann–Whitney, and chi-squared tests used.	Cachectic patients had significantly higher plasma IL-6 (*p* = 0.047) and IL-8 (*p* = 0.009), elevated CRP, higher CRP/albumin ratio, and lower albumin and hemoglobin compared to weight-stable patients. Gut barrier disruption evidenced by increased immune cell infiltration and altered cytokine expression. Findings support CRP, IL-6, and albumin as relevant biomarkers of cachexia progression.
Dolan et al. [22]	Retrospective exploratory study To examine the relationship between performance status, systemic inflammation, and cytokine profiles in advanced cancer.	40 adults with advanced cancer; 98% metastatic; median survival 91 days.	Retrospective analysis of baseline biomarkers including CRP, albumin, and 16 cytokines. mGPS and NPS calculated from routine blood values. Mann–Whitney U, Kruskal–Wallis tests, and Cox regression used for analysis.	IL-6 was the only cytokine consistently associated with both ECOG-PS/mGPS and ECOG-PS/NPS frameworks and poorer survival. Increasing ECOG-PS and mGPS were associated with higher IL-6, higher CRP, and lower albumin. Findings support IL-6 as a central cytokine mediator in tumor-host interactions and a potential therapeutic target in cancer cachexia.
Fearon et al. [5]	International Delphi consensus process To establish a foundational definition and classification framework for cancer cachexia.	No empirical patient data; expert consensus from oncologists, palliative medicine specialists, and nutritionists internationally.	Two-round Delphi process with focus groups; draft consensus statements rated anonymously on a 1–10 scale. Consensus defined as mean score ≥ 7. Literature review of cachexia studies from 1976–2010 informed the process.	Established CAC as ongoing skeletal muscle loss not reversible by nutritional support. Proposed three clinical stages: pre-cachexia, cachexia, and refractory cachexia. Defined diagnostic criteria as weight loss > 5%, BMI < 20 with >2% weight loss, or sarcopenia with >2% weight loss. Identified CRP as a key inflammatory biomarker and systemic inflammation as a central catabolic driver in CAC.
Go et al. [23]	Retrospective cohort study To examine the Cachexia Index (CXI) as a prognostic biomarker for survival in male SCLC patients.	267 male SCLC patients; limited-stage (*n* = 107) and extensive-stage (*n* = 160).	CXI calculated from pectoralis muscle SMI via CT scan at T4 level, serum albumin, and NLR. CXI cutoff determined by time-dependent ROC analysis. Kaplan–Meier survival analysis and Cox regression used for multivariate prognostic analysis.	Low CXI was independently associated with shorter PFS and OS, lower treatment response rates, and more frequent treatment discontinuation. Supports CXI, integrating albumin, SMI, and NLR as a clinically useful composite cachexia biomarker in lung cancer. Findings are limited to male patients only, restricting generalizability to female populations.
Gong et al. [12]	Retrospective cohort study To investigate the prognostic value of the Cachexia Index CXI with inflammation, nutrition, and survival in gastric cancer.	324 adults with gastric cancer; divided into high CXI (*n* = 161) and low CXI (*n* = 163) groups.	SMI measured from preoperative CT scans at L3 level. CXI calculated from SMI, serum albumin, and NLR. Serum CRP, IL-6, TNF-α, prealbumin, and albumin measured preoperatively. Univariate and multivariate Cox proportional hazards models and Kaplan–Meier survival curves with log-rank tests used.	Low CXI was significantly associated with advanced TNM stage, higher CRP and IL-6, lower albumin and prealbumin, and higher rates of postoperative pulmonary infections. Low CXI was an independent predictor of worse overall survival (HR 0.45, *p* < 0.001). Supports CXI as a useful composite prognostic biomarker integrating inflammation and nutritional status in gastric cancer.
Huang et al. [24]	Cross-sectional study To evaluate CXI as a biomarker for identifying cachexia and quality of life in gastric cancer patients.	431 adults; confirmed gastric cancer; 7 1.7% male and 28.3% female; median age 68 years; 38.52% met Fearon cachexia criteria and 37.12% met AWGC criteria.	CXI calculated from L3-SMI via CT scan, serum albumin, and NLR. Cachexia diagnosed using both AWGC and Fearon criteria. HRQoL assessed using EORTC QLQ-C30. ROC curves used to determine CXI cutoff values; univariate and multivariate logistic regression used.	CXI was significantly lower in cachectic versus non-cachectic patients. CXI was independently associated with AWGC-defined cachexia (OR = 0.98, *p* < 0.001). ROC analysis showed AUC of 0.752 for males and 0.717 for females. Low CXI was independently associated with poor HRQoL (OR = 1.79, *p* = 0.033), supporting CXI as a useful biomarker for identifying cachexia and poor quality of life in gastric cancer.
Li et al. [15]	Prospective multicenter cohort study To evaluate the Prognostic Nutritional CRP Ratio (NCR) as a survival predictor in cancer cachexia patients.	3447 adults with cancer cachexia; 66.6% male and 33.4% female; mean age 63.8 years; included lung, colorectal, gastric, esophageal, pancreatic, liver, and gynecological cancers.	NCR calculated as BMI × albumin/CRP; optimal NCR cutoff of 309.07 determined using maximally selected rank statistics. Cox proportional hazards regression used for univariate and multivariate survival analysis. Restricted cubic spline modeling assessed non-linear associations. Kaplan–Meier curves and log-rank tests used; quality of life assessed using EORTC QLQ-C30 and PG-SGA.	Lower NCR was significantly associated with higher all-cause mortality across multiple cancer types. Each standard deviation increase in NCR was associated with a 21–22% reduction in mortality risk. High NCR (≥309.07) was independently associated with significantly better overall survival (HR 0.56, *p* < 0.001). Findings support NCR as a comprehensive prognostic biomarker reflecting both nutritional status and systemic inflammation in cancer cachexia.
Liang et al. [22]	Retrospective cohort study To investigate the association between the Naples Prognostic Score (NPS) and cancer incidence and mortality using NHANES data (1999–2018).	42,582 adults; 4099 cancer survivors included mean age 47.74 years; 47.45% male and 52.55% female; cancer types included skin, urinary, breast, genital, digestive, and other cancers.	NPS calculated from serum albumin, total cholesterol, NLR, and lymphocyte-to-monocyte ratio. Patients stratified into three NPS groups (0, 1–2, 3–4). Cancer status determined by self-reported questionnaire; mortality data linked to National Death Index through December 2019. Multiple logistic regression, Cox proportional hazards models, and Kaplan–Meier survival analysis used.	Higher NPS was significantly associated with increased cancer incidence (OR 1.64, *p* < 0.05) and worse survival outcomes in cancer survivors. Compared to NPS Group 0, Group 2 had significantly higher all-cause mortality (HR 2.57), cardiovascular mortality (HR 3.44), and cancer-specific mortality (HR 1.60). Associations remained consistent across age, sex, race, and BMI subgroups. Supports NPS as a clinically useful composite index reflecting inflammation and nutritional status in cancer prognosis.
Lipshitz et al. [9].	Prospective case–control study To compare inflammatory biomarkers, quality of life, appetite, and cachexia between advanced cancer patients and healthy controls.	80 adults total; 40 patients with advanced stage 4 cancer patients, and 40 healthy age- and sex-matched controls.	Biomarkers assessed included albumin, hemoglobin, NLR, PLR, SII, CRP, TNF-α, IL-6, IL-8, CXCL5, and H3Cit. Quality of life assessed using EORTC QLQ-C30; appetite assessed using FAACT A/CS-12. Cachexia staged using a five-factor cachexia scoring system. ANOVA, Mann–Whitney, Pearson and Spearman correlations, and chi-square tests used.	Cachexia was present in 60% of cases and 30% reported poor appetite. Albumin, NLR, hemoglobin, PLR, SII, TNF-α, IL-8, and CRP were significantly elevated in cancer patients versus controls and showed significant relationships with all aspects of quality of life and cachexia severity. CXCL5 and H3Cit were not reliable cachexia biomarkers. Findings limited by small sample size, multiple primary diagnoses, and single private oncology setting.
Malla et al. [6]	Narrative review (supporting evidence) To examine the epidemiology and cytokine mechanisms of cancer cachexia.	No empirical patient data collected; synthesis of literature published after 2002.	Narrative literature review of English-language full-text papers published after 2002. Evidence synthesized from existing research on cytokine pathways, including IL-6 and TNF-α in cancer cachexia. No primary data collection or statistical analysis performed.	Cancer cachexia affects approximately half of cancer patients and accounts for one-quarter of cancer deaths. IL-6 and TNF-α drive muscle catabolism through NF-κB and JAK/STAT signaling pathways, promoting systemic inflammation, anorexia, and muscle wasting. Findings support IL-6 and TNF-α as central cytokine mediators in cancer cachexia pathophysiology, used to contextualize the inflammatory biomarker framework of this review.
Meyer-Knees, et al. [25]	Prospective cohort study (CONKO 020) To compare inflammatory biomarkers and nutritional assessments as survival predictors in patients with gastrointestinal and pancreatic cancers.	182 adults with inoperable gastrointestinal cancer; 120 with PDAC and 62 with others gastrointestinal adenocarcinomas; 56.7% male; median age 63.9 years.	CRP, IL-6, albumin, and hemoglobin measured at baseline and during follow-up. Conventional nutritional assessments, including BMI and bioelectrical impedance analysis (BIA) also performed. Kaplan–Meier survival analysis, log-rank tests, and univariate and multivariate Cox regression used for statistical analysis.	In PDAC patients, high CRP and IL-6 at diagnosis were significantly associated with worse overall survival. CRP was the only independent prognostic factor at diagnosis (HR 1.91, *p* = 0.003) and over the disease course (HR 2.21, *p* < 0.001). Low albumin was independently associated with worse survival over the disease course (HR 1.71, *p* = 0.030). BMI and BIA parameters were not prognostic at diagnosis; findings support CRP and albumin as earlier and more sensitive prognostic biomarkers than conventional nutritional assessments in pancreatic cancer cachexia.
Prado et al. [26]	Narrative review (supporting evidence) To examine anti-cytokine agents targeting IL-6 and TNF-α in cancer cachexia treatment.	No empirical patient data; synthesis of clinical trial evidence.	Narrative review of published Phase I, II, and III clinical trials evaluating anti-cytokine agents targeting IL-6 and TNF-α pathways in cancer cachexia. No primary data collection or statistical analysis performed.	IL-6 and TNF-α drive cachexia pathogenesis. Multi-target anti-cytokine agents showed more promise than single-cytokine interventions. No approved pharmacological therapies currently exist. Future trials should target multiple cytokine pathways in earlier disease stages.

Note: CRP = C-reactive protein; IL-6 = Interleukin-6; TNF-α = Tumor necrosis factor-alpha; NLR = Neutrophil-to-lymphocyte ratio; CXI = Cachexia Index; NPS = Naples Prognostic Score; SMI = Skeletal muscle index; PDAC = Pancreatic ductal adenocarcinoma; NHANES = National Health and Nutrition Examination Survey; OS = Overall survival; HR = Hazard ratio; OR = Odds ratio.

**Table 2:** Summary of Biomarkers.

Biomarker	Muscle Loss	Quality of Life	Mortality Risk	Prognostic Index Use	Evidence Consistency
↑ **CRP**	**Strong**	**Strong** Association anorexia and hypermetabolism	**Strong**	CXI; Asian working group for cachexia criteria)	Very High (Validated prognostic marker across cancer types)
↑ **IL-6**	**Strong**	**Strong** ↑ Fatigue ↓ Muscle strength	**Strong**	ELISA/Multiples Assays; not yet included in cachexia indices.	High, targeted in anti-cytokine therapeutic research (IL-6 inhibitors, i.e., tocilizumab), but not part of validated cachexia scoring tool.
↑ **TNF-α**	**Moderate**	**Moderate** Metabolic dysfunction, anorexia, and systemic inflammation	**Moderate**	ELISA; not part of validated indices but formative in multi-biomarker panels	Moderate (Mechanistic relevance; limited index integration)
↑ **NLR**	**Moderate**	**Moderate** Linked to inflammation, muscle loss, and poor physical function	**Moderate**	CBC, CXI, NPS	NLR is one of the most practical inflammatory components
↓ **Albumin**	**Moderate**	**Moderate** Associated with anorexia, inflammation, and survival	**Moderate**	Comprehensive Metabolic Panel (CMP) or Serum Albumin	High (Consistently used in validated nutritional-inflammatory indices)

CRP: C-reactive protein; IL-6: Interleukin-6; TNF-α: Tumor necrosis factor-alpha; CBC: Complete blood count; CXI: Cachexia Index: NPS: Naples Prognostic Score; NLR: Neutrophil-Lymphocyte Ratio; ↑: Increase; ↓: Decrease.

## Data Availability

No new data were created or analyzed in this study. Data sharing is not applicable to this article.
